# *In Vivo* Anti-Diabetic Activity of the Ethanolic Crude Extract of *Sorbus decora* C.K.Schneid. (Rosacea): A Medicinal Plant Used by Canadian James Bay Cree Nations to Treat Symptoms Related to Diabetes

**DOI:** 10.1093/ecam/nep158

**Published:** 2011-08-11

**Authors:** Rose Vianna, Antoine Brault, Louis C. Martineau, Réjean Couture, John T. Arnason, Pierre S. Haddad

**Affiliations:** ^1^CIHR Team in Aboriginal Anti-Diabetic Medicines, Université de Montréal, Montreal, QC, Canada H3T 1J4; ^2^Natural Health Products and Metabolic Diseases Laboratory, Department of Pharmacology, Université de Montréal, Montreal, QC, Canada H3T 1J4; ^3^Department of Physiology, Université de Montréal, Montreal, QC, Canada H3T 1J4; ^4^Department of Biology, University of Ottawa, ON, Canada

## Abstract

A number of potential anti-diabetic plants were identified through an ethnobotanical survey of the traditional pharmacopeia of the Cree of Eeyou Istchee (CEI—Northeastern Canada) used against symptoms of diabetes and their biological activity assessed by *in vitro* bioassays. Among these, *Sorbus decora* C.K.Schneid. (Rosacea) ranked highly and increased the transport of glucose in skeletal muscle cells in culture. The present study thus aimed at confirming the antidiabetic potential of *S. decora* in *in vivo* models of insulin resistance and diabetes, notably the streptozotocin Type 1 diabetic rat (STZ), the genetic KK-A^y^ Type 2 diabetic mouse and the rat rendered insulin resistant with 10% glucose water consumption for 6 weeks. *Sorbus decora* ethanolic crude extract (SDEE) was administered orally (200 mg kg^−1^) and compared to metformin (150 or 500 mg kg^−1^). The intragastric (i.g.) gavage of SDEE transiently decreased glycemia in STZ rats in a bi-phasic manner but the effect was cumulative over several days. In KK-A^y^ mice, SDEE incorporated in food (0.12%) decreased glycemia by 15% within 1 week as compared to vehicle controls. In pre-diabetic insulin-resistant rats, SDEE fed daily by i.g. gavage for 2 weeks significantly decreased the slight hyperglycemia and hyperinsulinemia, without affecting sugar water intake. Using the HOMA insulin resistance parameter, the effect of SDEE was equivalent to that of metformin. In conclusion, the ethanolic crude extract of *S. decora* demonstrates both anti-hyperglycemic and insulin-sensitizing activity *in vivo*, thereby confirming anti-diabetic potential and validating CEI traditional medicine.

## 1. Introduction

Diabetes mellitus is a metabolic disease characterized by hyperglycemia resulting from defects in insulin secretion, insulin action or both. Type 1 diabetes is caused by a deficiency of insulin secretion from *β*-pancreatic cells. On the other hand, Type 2 diabetes is closely associated with obesity and is characterized by an initial phase of progressive insulin resistance, with an ensuing reduction in the ability of the pancreatic hormone to promote peripheral glucose disposal and to suppress hepatic glucose output [1]. Sedentary life style, unhealthy dietary habits and genetic predisposition are some of the key factors that have conspired to create the current worldwide epidemic of Type 2 diabetes, an acquired syndrome of elevated blood glucose. In 2005, 246 million people worldwide suffered from Type 2 diabetes and this number is projected to grow to 366 million by 2030 [2], partly due to increased obesity. Aboriginal populations worldwide, such as the Cree of Eeyou Istchii (CEI—from James Bay region of the Canadian province of Quebec), are particularly at risk. Indeed, CEI communities are more affected by obesity (30.1% versus 14.0%) and Type 2 diabetes (14.5% versus 4.1%) than the rest of Canada [3]. Compounding the lifestyle changes and genetic predisposition, modern medicine and its therapeutic arsenal are disconnected from CEI culture. Drug treatment compliance is thus less than optimal and diabetes complications therefore also more prevalent [3]. In an effort to develop culturally adapted complementary and alternative therapies based on CEI traditional medicine, our Team has identified several medicinal plants of the Boreal forest used to treat several symptoms of diabetes [4]. A number of these were confirmed to have significant anti-diabetic potential when screened using *in vitro* bioassays [5]. Among these, the ethanolic crude extract of *Sorbus decora* C. K. Schneid. (Rosacea) showed effectiveness in increasing glucose uptake in skeletal muscle cells (C2C12) [5]. The aim of this study was to confirm the anti-diabetic activity of *S. decora in vivo*. We used three animal models of *diabetes mellitus*, in order to comprehensively evaluate the impact of the ethanolic crude extract of *S. decora* on several pathological states related to diabetes. Namely, Type 1 diabetes was induced by *streptozotocin* (STZ), a nitrosurea derivative isolated from *Streptomyces achromogenes* able to induce specific destruction of *β*-pancreatic cells in rats, thus representing a typical model of insulin dependence. Second, we studied effects of *S.decora* in a pre-diabetic insulin resistance model, induced in rats with a 10% glucose solution as drinking water [6, 7]. Finally, we used the Type 2 diabetic KK-A^y^ mouse, a hyperphagic obese strain that develops insulin resistance, compensatory hyperinsulinemia and islet cell hyperplasia [8].

## 2. Methods

### 2.1. Animals

Male Sprague-Dawley (SD) rats (weighting 200–250 g) were obtained from Charles River (St Constant, Québec, Canada), housed 1 per cage and allowed 1 week to adapt to their new environment. The KK-A^y^ mice were obtained from an in-house colony developed from breeding pairs acquired from Jackson Laboratories (Bar Harbour, ME, USA) and housed individually. The animals were maintained in an environment of controlled temperature (20°C) and humidity (53%) under a 12 h light-dark cycle. For rats, standard rodent chow and water were provided *ad libitum* throughout the experimental period. KK-A^y^ mice were treated similarly except during the treatment period, when they received the plant incorporated in the chow (see “*in vivo* treatment administration” below). All animal procedures used were in strict accordance with the Canadian Council on Animal Care *Guide to the Care and Use of Experimental Animals* and all experimental protocols were approved by the Université de Montréal animal experimentation ethics committee.

### 2.2. Plant Material

The plant *S. decora* C.K. Schneid. (Rosaceae) was harvested in Mistissini, Quebec, Canada, as previously described [5] and by following the instructions of the elders and healers of this community. The identification of the plant was confirmed by taxonomist Dr A. Cuerrier and a specimen was deposited in the Marie-Victorin herbarium of the Montreal Botanical Garden, Montreal, Quebec, Canada (number: MIS 03-9).

### 2.3. Preparation of the Ethanolic Crude Extract

The plant was air dried at the University of Ottawa and inner bark was extracted twice with 10 ml of 80% ethanol per gram for dry material on a mechanical shaker for 24 h, and then filtered using Whatman paper. The first and the second extracts were combined and dried in a rotatory evaporator followed by lyophilization and preserved at 4°C and protected from light.

### 2.4. Induction of *Diabetes Mellitus*

Type 1 diabetes was induced by a single intraperitoneal (i.p.) injection of 100 *μ*l of sterile phosphate buffered solution (PBS—pH 7.4) containing *streptozotocin* (STZ) (65 mg kg^−1^), (Zanosar, Pharmacia & Upjohn, ON, Canada) and after 4 days the hyperglycemia was established [9]. Glucose concentration was measured in a blood sample obtained from tail puncture, with a glucose oxidase-impregnated test strip and a reflectance meter (Accu-Check III, Boehringer Mannheim, Germany). Only animals that had a blood glucose concentration higher than 20 mM 4 days after treatment with STZ were used for the study [9], generally >80% of STZ treated animals. Control rats were injected with PBS only.

### 2.5. Induction of Insulin Resistance

Insulin resistance was induced as detailed by El Midaoui [6, 7] with some modifications. The insulin-resistant group of animals was given a solution of 10% d-glucose as drinking water and a normal chow diet during 6 weeks. Control animals were fed for 6 weeks with tap water and normal chow diet. At the end of the treatment period, blood was collected for the subsequent measurement of glucose and insulin in animals fasted for 16 h. The plasma glucose concentration was measured with a glucometer (Accu-Check III, Boehringer Mannheim, Germany) and insulin levels were determined by radioimmunoassay method (Rat Insulin RIA kit, Linco Research, St Charles, MO, USA). To evaluate the degree of insulin resistance, the Homeostasis Model Assessment (HOMA) was used as an index of insulin resistance and calculated by the following formula: insulin (*μ*U ml^−1^) × glucose (mol l^−1^)/22.5 [6, 7]. Only animals that demonstrated a significant level of insulin resistance after 4 weeks of the 10% glucose water treatment were selected for the study (generally 80% of the treated animals).

### 2.6. KK-A^y^—Genetic Model of Type 2 Diabetes

The insertion of the yellow agouti gene (Ay) into KK mouse strain results in a congenic yellow obese KK mouse strain, the KK-A^y^ mice. It represents a genetic model of Type 2 diabetes that exhibit severe obesity, hyperinsulinemia and insulin resistance, all cardinal features of Type 2 diabetes [10].

### 2.7. *In Vivo* Treatment Administration

Preliminary experiments were carried out with several vehicles to administer the crude plant extract to animals, including 80% ethanol or a mixture of 5% Tween and 5% ethanol in distilled water. The latter two vehicles gave optimal results. In rats, *S. decora* ethanolic crude extract (200 mg kg^−1^) was administered by intra-gastric gavage, either as a single dose (acute treatment) or as a repeated daily administration over 7–14 days (chronic treatment), as reported by others [11]. Metformin was used as a positive control at doses determined to be optimal in preliminary studies (500 mg kg^−1^ in STZ rats and 150 mg kg^−1^ in insulin-resistant rats) and was also administered by intragastric (i.g.) gavage in conditions identical to *S. decora*-treated congeners. Appropriate vehicle controls were also used in parallel respecting strictly identical experimental protocols. Because i.g. gavage is more difficult and stressful for mice, KK-A^y^ animals received *S. decora* ethanolic crude extract mixed in powdered rodent chow at 0.12% and reconstituted into pellets. This dose was determined to be equivalent to 200 mg kg^−1^ based on the measured daily food consumption of the animals.

### 2.8. Statistical Analysis

Results are given as means ± SEM. The significance of differences between means was evaluated by Student's *t*-test or one-way analysis of variance (ANOVA), as appropriate, followed by *post hoc* Neuwman-Keuls analysis using StatView software version 4.01 (Cary, NC, USA). *P*-values < 0.05 were considered to be statistically significant.

## 3. Results

### 3.1. *Sorbus decora* Reduces Glycemia in Streptozotocin Type 1 Diabetic Rats

**Figure 1:**
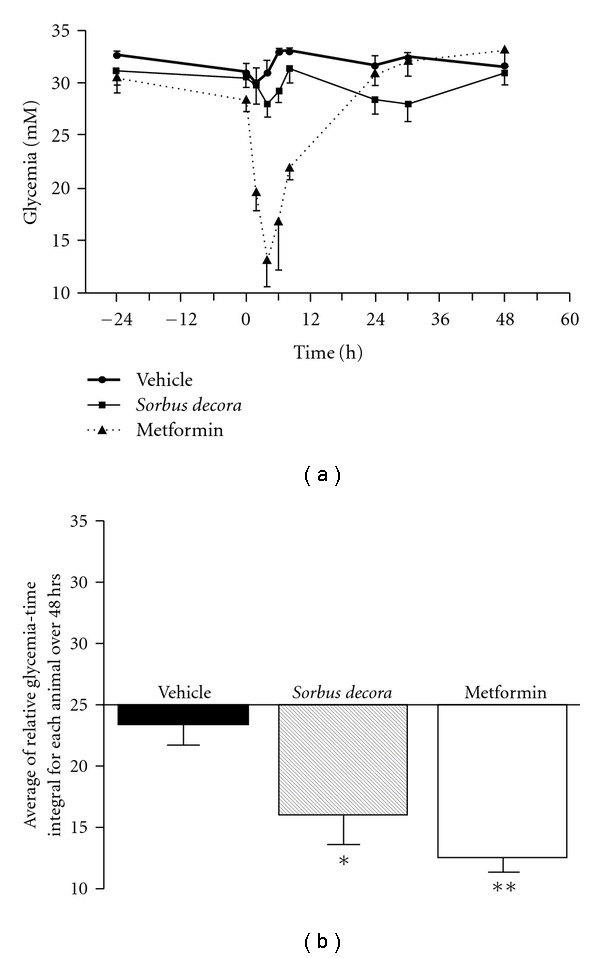
*Sorbus decora* extract acutely decreases glycemia in Type 1 diabetes in a bi-phasic manner. (a) Glucose concentrations were measured in the plasma of STZ diabetic rats pre-treated orally with vehicle (80% ethanol in distilled water), *S. decora* (200 mg kg^−1^) or metformin (500 mg kg^−1^). (b) The area above the glycemia versus time curve was assessed relative to initial glycemia used as the baseline reference. Data are presented as means ± SEM (*n* = 6). Significantly different from the vehicle-treated group, *P < .05 or **P < .01

**Figure 2:**
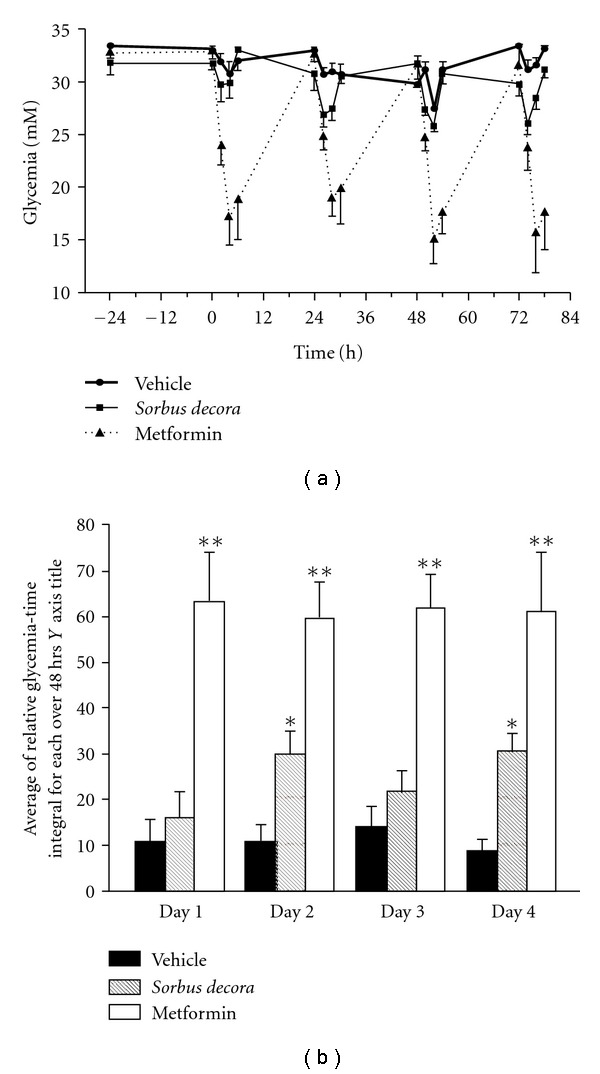
Effect of repeated oral treatment with *S. decora* ethanolic crude extract on glycemia in Type 1 diabetes: evidence of a cumulative effect. (a) Glucose concentrations were measured in the plasma of STZ diabetic rats treated orally in a repeated daily manner with vehicle (80% ethanol in distilled water), *S. decora* (200 mg kg^−1^) or metformin (500 mg kg^−1^). (b) The area above the glycemia versus time curve was assessed relative to initial glycemia used as the baseline reference. Data are presented as means ± SEM (*n* = 6). Significantly different from vehicle-treated group, *P < .05 or **P < .01.

We first used streptozotocin (STZ)-treated rats to assess the hypoglycemic activity of the crude ethanolic extract of *S. decora* (200 mg kg^−1^, i.g.) in this Type 1 diabetes setting. As shown in Figure 1(a), a single i.g. administration of *S. decora* extract significantly decreased glycemia in a bi-phasic manner with peaks at 4 h (9.7 ± 3.7% inhibition) and 30 h (14.4 ± 4.2% inhibition) as compared with vehicle-treated animals. The 80% ethanol vehicle also yielded a small and transient decrease in glycemia similar to that seen for the initial peak observed with *S. decora* ethanolic crude extract. However, its extent was significantly lower than that of *S. decora* and no second decrease was observed. This was confirmed by measuring the area above the glycemia versus time curve using initial glycemia as the baseline reference (Figure 1(b)). As a positive control, we used the oral hypoglycemic drug metformin (optimal dose of 500 mg kg^−1^ in this model, i.g.); it induced a dramatic drop in STZ rat glycemia that was monophasic and also peaked at 4 h. We next evaluated the impact of repeated daily gavage of our treatments on STZ rat glycemia. As shown in Figure 2(a), daily *S. decora* ethanolic crude extract treatment yielded the expected initial transient decrease in plasma glucose just described. However, as treatment days advanced, peak decreases in glycemia gradually reached greater intensity, suggesting a cumulative effect. In contrast, repeated metformin treatment yielded similar large decreases in STZ rat glycemia irrespective of the number of days of i.g. administration. This was confirmed by the area above the curve analysis (Figure 2(b)).

### 3.2. *Sorbus decora* Improves Insulin Sensitivity in Insulin-Resistant Rats

**Figure 3:**
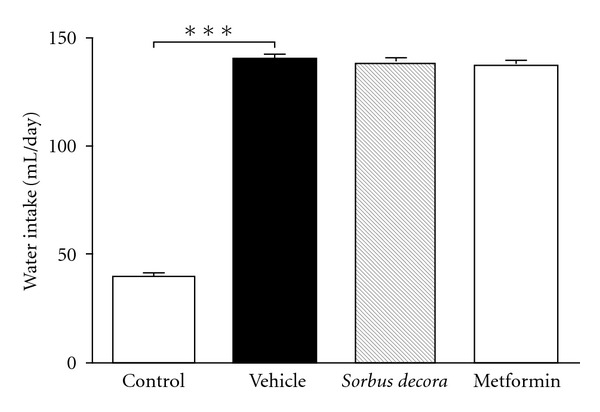
Effect of *S. decora* ethanolic crude extract on water intake in glucose-fed rats. Water intake was measured daily in rats fed with a solution of 10% glucose or tap water (control) for 6 weeks and treated daily with an oral administration of either vehicle (black bar), *S. decora* (200 mg kg^−1^) or metformin (150 mg kg^−1^) during the last 2 weeks of treatment. Data are presented as means ± SEM (*n* = 6). Significantly different from vehicle treated animals, ***P < .001.

**Figure 4:**
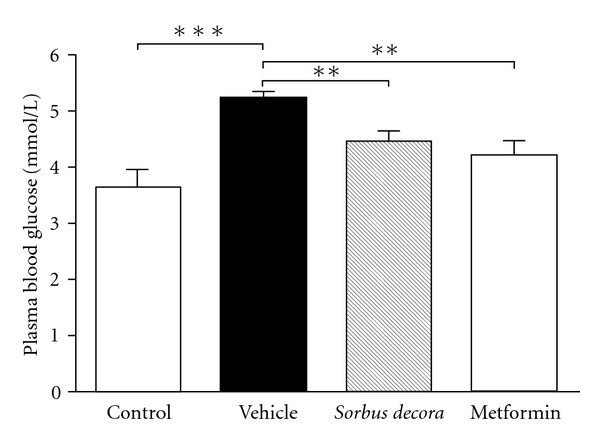
Effect of *S. decora* ethanolic crude extract on glycemia in glucose-fed rats. Glucose concentrations were measured in the plasma of rats fed with a solution of 10% glucose or tap water (control) for 6 weeks and treated daily with an oral administration of either vehicle (black bar), *S. decora* (200 mg kg^−1^) or metformin (150 mg kg^−1^) during the last 2 weeks of treatment. Data are presented as means ± SEM (*n* = 6). Significantly different from vehicle-treated group, **P < .05 or ***P < .01.

**Figure 5:**
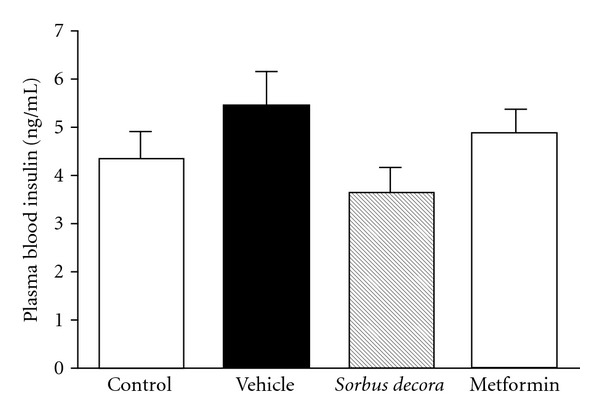
Effect of *S. decora* extract on insulinemia in glucose-fed rats. Insulin concentrations were measured by RIA in the plasma of rats fed with a solution of 10% glucose or distilled water (control) for 6 weeks and treated daily with an oral administration of either vehicle (black bar), *S. decora* (200 mg kg^−1^) or metformin (150 mg kg^−1^) during the last 2 weeks of treatment. Data are presented as means ± SEM (*n* = 6).

**Figure 6:**
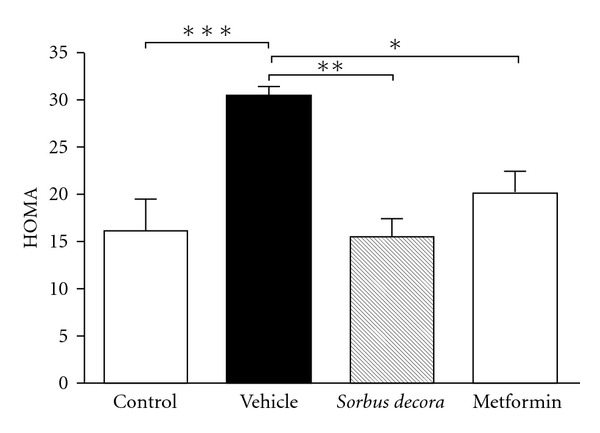
Effect of *S. decora* extract on insulin resistance in glucose-fed rats. The homeostasis model assessment of insulin resistance (HOMA) were measured in the plasma of rats fed with a solution of 10% glucose or tap water (control) for 6 weeks and treated daily with an oral administration of either vehicle (black bar), *S. decora* (200 mg kg^−1^) or metformin (150 mg kg^−1^) during the last 2 weeks of treatment. Data are presented as means ± SEM (*n* = 6). Significantly different from vehicle-treated group, *P < .05, **P < .01 or ***P < .001.

The second animal model used to assess *S. decora* anti-hyperglycemic potential *in vivo* was the rat insulin resistance model based on chronic treatment with d-glucose (10%) in drinking water. As previously reported [6, 7], chronic glucose water feeding more than doubled the daily water intake (milliliters per day) as compared to rats receiving daily i.g. administration of vehicle (5% ethanol, 5% Tween 80 in distilled water—Figure 3). Moreover, daily treatment with ethanolic crude extract of *S. decora* (200 mg kg^−1^ day^−1^, i.g.) or metformin (optimal dose of 150 mg kg^−1^ day^−1^ in this model, i.g.) for the last 2 weeks of a 6-week chronic glucose water-feeding regimen did not affect daily water consumption. Effects of the treatment regimens on fasting blood glucose are presented in Figure 4. Chronic glucose water feeding resulted in a modest but statistically significant increase in glycemia as expected in this insulin-resistant model [6, 7]. This modest hyperglycemia was effectively and similarly normalized by either *S. decora* or metformin treatments (Figure 4). This insulin-resistant model is also characterized by modest hyperinsulinemia [6, 7], as observed herein in vehicle-treated animals compared to normal non-glucose-fed controls (Figure 5). A 2-week treatment with the ethanolic crude extract of *S. decora* was effective in reducing insulinemia below the level of non-glucose-fed controls. Metformin brought circulating insulin levels back toward levels found in non-glucose-fed rat congeners. However, data variability prevented the assignment of statistical significance to these findings (ANOVA NS). Nonetheless, when the homeostasis model assessment (HOMA) insulin-resistance index was evaluated, results were very convincing. Indeed, as expected, chronic glucose feeding resulted in a large and significant increase in HOMA values as compared to normal water-fed rats (Figure 6). Treatment with both the ethanolic crude extract of *S. decora* and metformin was effective in reducing HOMA values back to levels observed in normal animals, with a tendency for the plant extract to have a greater effect than the reference oral hypoglycemic drug (Figure 6), thereby confirming results on glycemia and insulinemia.

### 3.3. Effects of *S. decora* in KK-A^y^ Type 2 Diabetic Mice

**Figure 7:**
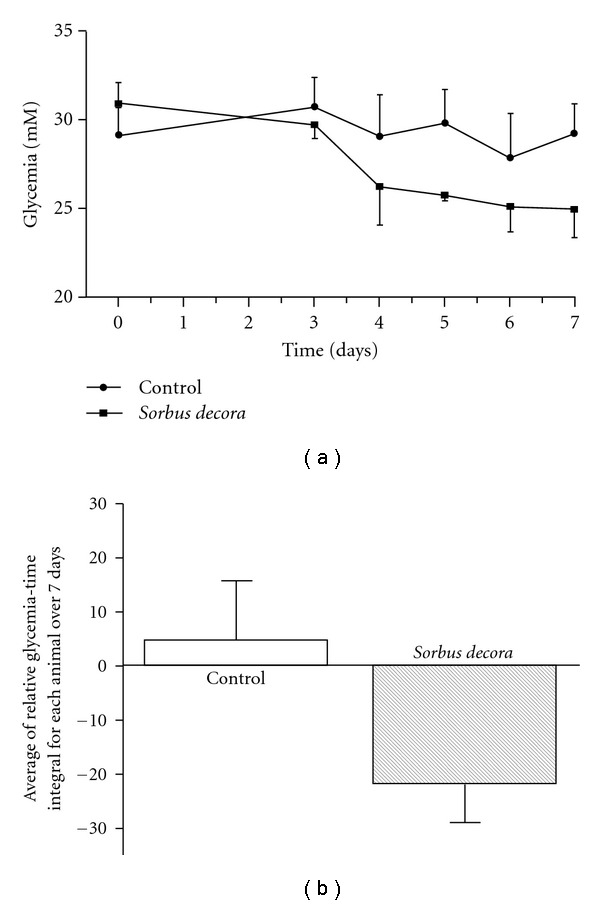
Effect of chronic treatment with *S. decora* extract in Type 2 diabetic KK-A^y^ mice. (a) Blood glucose concentrations were measured in animals receiving regular chow (control) or *S. decora* mixed in chow at 0.12%. (b) The area below or above the glycemia versus time curve was assessed relative to initial glycemia used as the baseline reference. Data are presented as means ± SEM (*n* = 6).

Lastly, we used the KK-A^y^ genetically obese mouse as a model of full-blown hyperglycemic Type 2 diabetes. Diabetic KK-A^y^ mice received the ethanolic crude extract of *S. decora* mixed in food at 0.12% (to deliver 200 mg kg^−1^ day^−1^) during 7 days. Incorporation into food was chosen over i.g. administration in order to avoid the larger stress of gastric administration in these smaller animals. Blood glucose levels decreased in *S. decora*-treated mice within 4 days of the onset of treatment (Figure 7) and remained depressed thereafter. In comparison, control KK-A^y^ fed normal laboratory chow exhibited a stable glycemia over the 7-day experimental period.

## 4. Discussion

The Cree people of Eeyou Istchee (CEI) in northern Quebec, like most other North American tribes, have faced significant and rapid changes in their lifestyle including reduced physical activity and increased consumption of non-traditional foods [12, 13]. As a consequence, obesity and diabetes are now highly prevalent in CEI, whereas they were much less frequent only 25 years ago. Indeed, although the life expectancy of First Nations people is still the same as the whole of Canada (75.5 and 77.7 years), the CEI population is 2.15 times more obese and has 3.5 times more diabetic than non-aboriginals in Canada [3]. Aside from genetic and environmental factors, the cultural disconnect of modern pharmaceutical interventions leads to less compliance to diabetes treatments and consequently greater rates of diabetes complications, notably diabetic neuropathy and nephropathy [3]. Multimodal interventions, most notably those that are culturally better adapted, are therefore urgently needed.

To address this, a multidisciplinary community-based project was put in place joining three Canadian universities (Montreal, McGill and Ottawa) with the Cree Board of Health and Social Services and a number of Cree communities in an effort to explore the potential benefit of including Boreal forest plants stemming from Cree traditional medicine as part of such multimodal interventions. A novel ethnobotanical approach based on diabetes symptoms successfully identified potential anti-diabetic plants [4], whose biological activity was confirmed by a platform of antidiabetic cell-based bioassays [5]. Among the most promising plants was *S. decora*. Indeed, *in vitro* studies demonstrated that the ethanolic crude extract of the *S. decora* was able to increase glucose uptake in muscle cells and to protect preneuronal PC12 cells against high glucose conditions, highlighting the anti-diabetic properties for *S. decora* [5]. In the present study, we used three different diabetic experimental models (pre-diabetic, Types 1 and 2 diabetes) to confirm *in vivo* the anti-diabetic activity demonstrated by *S. decora* from *in vitro* assays.

The rat pre-diabetic model chosen is characterized by modest hyperinsulinemia and hyperglycemia secondary to insulin resistance [6, 7]. Our results clearly showed that daily i.g. treatment with the ethanolic crude extract of *S. decora* during last 2 weeks of the 6-week glucose feeding regimen gave results equivalent to the reference oral hypoglycemic drug metformin. Indeed, both treatments significantly reduced glycemia as well as the HOMA insulin resistance index without affecting glucose water intake or body weight.

Similar encouraging results were obtained in the streptozotocin-treated rat (4 days), a model of Type 1 diabetes. This model is much more severe with circulating glucose in the 25–30 mM range and severely deficient pancreatic *β*-cell function [9]. Despite the severity of the disease, the acute administration of the crude ethanol extract of *S. decora* succeeded in significantly reducing blood glucose in a bi-phasic manner by 10%–15%. When such administration was repeated over several days, a cumulative effect was suggested. In comparison, metformin was much more potent at reducing the glycemia of STZ rats, but it did so in a monophasic manner with no evidence of a cumulative effect over the experimental period used.

Finally, we used the KK-A^y^ mouse model that is characterized by obesity and expresses severe Type 2 diabetes (glycemia in the range of 20–25 mM). Because mice are more sensitive to needle i.g. gavage (stress having a major impact on glycemia in these animals; Brault, Martineau and Haddad, unpublished observations), we incorporated the ethanolic crude extract of *S. decora* into the diet of the animals. Over a 7-day period, glycemia had a tendency to diminish but data variability prevented the assignment of statistical significance to this hypoglycemic effect. It is possible that the dose was not sufficiently high, even though it was calculated to be equivalent to the dose delivered to rats by i.g. gavage on a daily basis. It must also be considered that rats and mice may not express the same sensitivity to the plant extract. Alternatively, bioavailability may have been lower in the rodent chow matrix than in the solvents used for gavage, especially since the intake of the extract was spread over the feeding period as opposed to the bolus dose provided by i.g. gavage. However, in our hands, metformin administered through the diet completely normalizes glycemia in KK-A^y^ mice over a 3-4-day period, as observed elsewhere [14]. Nevertheless, it remains possible that the treatment period with *S. decora* ethanolic crude extract was not sufficiently long for glycemia to reach statistically lower levels, as compared to control mice that had a very stable glycemia over the 7-day regimen. Future studies should thus address these considerations to confirm the hypoglycemic action of *S. decora* ethanolic crude extract in this animal model.

Recent studies in our laboratory indicate that *S. decora* acts by a mechanism similar to that of metformin. Metformin is a dimethylbiguanide oral hypoglycemic drug derived from guanidine, a hypoglycemic active compound isolated from *Galega officinalis* [15] or French Lilac, a medicinal plant used for centuries in Europe for diabetes treatment. Metformin is widely prescribed for humans with Type 2 diabetes in the world [15]. It is able to inhibit hepatic glucose production and also acts as an insulin sensitizer in isolated skeletal muscle from insulin-resistant humans [16]. It was demonstrated that the enzyme AMP-activated kinase (AMPK) is activated by metformin [15–19]. AMPK is a key sensor of cell energetic balance being activated by increase in the ratio AMP/ATP. There are indications that the activation of this enzyme is beneficial for the treatment and prevention of Type 2 diabetes and the metabolic syndrome [19–21]; its activation leads to increased glucose uptake in rat skeletal muscle [22]. Likewise, the ethanolic crude extract of *S. decora* increases glucose uptake in C2C12 cells by means of AMPK activation, which appears to implicate the inhibition of mitochondrial respiration [23]. These cellular and molecular actions may thus play a significant role in the hypoglycemic activity of the plant *in vivo*.

In summary, taken together, all *in vivo* models of insulin resistance and diabetes used herein confirm the anti-diabetic potential of *S. decora*, with a caveat that greater effects may be obtained in less severe stages of the disease. The present studies, therefore, provide compelling scientific evidence that plants used within the traditional medicine of the CEI to treat diabetic symptoms do possess significant antidiabetic potential in animal models of diabetes. Plants such as *S. decora* are currently used by Cree populations of Mistissini without notable signs of toxicity (S. Grandi et al., unpublished observations). Nonetheless, clinical studies are required to assess the potential benefit of using *S. decora* preparations in humans. Our studies pave the way to such studies and support the inclusion of such traditional medicine as part of a multimodal set of interventions to help Cree diabetics to manage their disease in a culturally relevant manner.
